# Monthly Report of Deaths in Buffalo for August, 1851

**Published:** 1851-10

**Authors:** W. L. G. Smith

**Affiliations:** Clerk


*Monthly Report of Deaths in Buffalo for August,* 1851.

Board of Health, *Buffalo*, Sept., 1851.

Number of deaths reported to the Board of Health in the month of August last:—Males, 92; females, 63 — total, 156. Age — Adults, 42; children, 114. Diseases — Consumption, 18; scarlet fever, 2; small pox, –; typhus fever, 8; pneumonia, 6; dysentery, 23; cholera infantum, 1; measles, 1; paralysis, 1; summer complaint, 18; unknown, 7; cholera, 3; pricarditis, 2; tetanus, 1; chronic inflammation of brain, 1; cholera morbus, 1; syphilis, 1; convulsions, 10; disease of the liver, 2; inflammation of the bowels, 2; inflammation of the brain, 3; hemorrhage of the bowels, 1; gastritis, 1; marasmus, 1; teething, 2; hydrocephalus, 3; old age, 2; still-born, 4; whooping cough, 8; casualty, 2; in child bed, 1.

By order of the Board,

W. L. G. SMITH, Clerk.

